# Late-life depression in sub-Saharan Africa: lessons from the Ibadan Study of Ageing

**DOI:** 10.1017/S2045796020000578

**Published:** 2020-07-20

**Authors:** A. Ojagbemi, T. Bello, O. Gureje

**Affiliations:** Department of Psychiatry, College of Medicine University of Ibadan, Ibadan, Nigeria

**Keywords:** Epidemiological transition, low-/middle-income countries, social determinants, years lived with disability

## Abstract

The population surviving to old age in sub-Saharan Africa (SSA) is increasing rapidly in consonance with the rest of the world. Nevertheless, the sub-region offers unique challenges to survival across the lifespan. The determinants of health and ageing in SSA are thus likely different from those in higher income countries. The need to explore pressing epidemiological and health service challenges of older people living in SSA in the context of multiple social changes and rapid ageing of the population provided the rationale for the Ibadan Study of Ageing (ISA). This article appraises ISA findings in relation to late-life depression. It concludes that healthcare policies in SSA need to deliberately prioritise the treatment of depression and other mental health problems in late-life in order to stem the neglect of older people's mental health in the region.

## Introduction

The population surviving to old age is increasing rapidly in all regions of the world. The projection is that by 2050 approximately 80% of persons who will be ⩾60 years globally will be residents of low-/middle-income countries (LMICs), with approximately 212 million living in Africa (Dotchin *et al*., 2013). As with physical health, the burden of mental health conditions increases with age (World Health Organization, 2017).

Mental health conditions rank among the leading causes of years lived with disability (Eaton *et al*., 2011), and depression is the most common and disabling mental health condition after the age of 60 years (Whiteford *et al*., 2013). Late-life depression is associated with complex comorbidities and chronic course of symptoms and disability (Haigh *et al*., 2018; Ojagbemi *et al*., 2018*a*). The disability-weight ascribed to late-life depression is set to increase in the coming few years (Whiteford *et al*., 2013), with some observational studies (Gureje *et al*., 2011) suggesting that compared with higher income countries (HICs), the disability-burden of late-life depression may be much higher in LMICs such as those in much of sub-Saharan Africa (SSA).

In contrast to reports from elsewhere (Savva *et al*., 2013; Darker *et al*., 2016), the social determinants of late-life depression in SSA may be different from those in HICs. In particular, the social, economic and health challenges faced by people living in SSA may present unique risks for the occurrence of depression among older people. For example, poverty, social deprivation, a high burden of infectious disease and a rapidly growing burden of non-communicable diseases in the sub-region (Cappuccio and Miller, 2016; Ojagbemi *et al*., 2017*b*) are potential risks for depression in old age. Major social changes in SSA, including those related to migration and urbanisation as well as the changing roles of women, are leading to the erosion of traditional multigenerational living arrangement. These factors, combined with the effects of globalisation, are changing the status of older people in the sub-region (United Nations, 2017; Ojagbemi and Gureje, 2019*b*) and stripping away traditional support systems. Older persons may be less equipped to cope with these challenges (Leigh-Hunt *et al*., 2017; The Academy of Medical Sciences, 2018) and become increasingly exposed to social isolation and its effects (especially depression) (Ojagbemi and Gureje, 2019*a*; Ojagbemi and Gureje, 2019*b*). Furthermore, there are no formal social welfare packages for older people in many countries in SSA and access to healthcare is often limited and dictated by personal financial resources (Uwakwe *et al*., 2009). The need to explore these pressing epidemiological, social and health challenges of older people living in SSA provided the rationale for the Ibadan Study of Ageing (ISA).

### The Ibadan Study of Ageing

The ISA comprised two integrated components. An initial cross-sectional study that was conducted to determine the profile and availability of informal care for older persons in need of care was followed by a 5-year prospective observation of the same cohort to investigate key determinants of successful ageing in the population. The integration of both phases allowed for the examination of both cross-sectional and longitudinal relationships among social, psychological and physical health indices. The main hypothesis of the ISA was that factors reflecting social changes associated with the adoption of ‘modern’ lifestyles will be associated with ageing outcomes in Nigeria.

The ISA cohort comprised persons who were 65 years or older living in households within communities spread across the Yoruba-speaking south-west and north-central regions of Nigeria (or nearly a quarter of the Nigerian population at the time of the study). Notably, Nigeria is the African country with the largest population. Selection of respondents was done using a multistage cluster sampling of local government areas (LGAs), followed by enumeration areas (geographical units demarcated by the National Population Commission) within the LGAs, and households in the enumeration areas.

Households included in the ISA were randomly generated from a computer listing of all eligible households in the selected enumerated areas. When the household had more than one eligible person, the eligible respondent per household was selected using the Kish method (Kish, 1949). In cases where the person so selected refused to participate, no replacement was chosen in the same household.

National representativeness of the ISA sample was achieved through the application of poststratification sampling weights in accordance with the stratified multistage sampling procedures (Bell *et al*., 2012). Common sources of sampling bias (e.g., non-response and chance) were examined by comparing age and gender categories with those of the Nigerian national population while adjusting for differences between the sample and the total Nigerian population (Brick and Kalton, 1996). The ISA is thus the largest community-based prospective observation of the health and wellbeing of older persons in SSA.

The highly successful study has produced over 30 publications which have generated a wealth of information about the mental health of older people living in SSA. This article appraises ISA findings and lessons learnt in relation to late-life depression.

## Late-life depression in the ISA

Major depressive disorder (MDD) was assessed with the fully structured World Health Organization (WHO) Composite International Diagnostic Interview (CIDI) (Kessler *et al*., 2004). The diagnosis was made on the basis of the criteria of the Diagnostic and Statistical Manual of Mental Disorders, fourth edition (DSM-IV) (American Psychiatric Association, 1994).

Additional quantitative assessment of depression symptoms was conducted using the 30-item Geriatric Depression Scale (GDS) (Yesavage *et al*., 1982). The GDS was developed specifically for use in older populations (Yesavage *et al*., 1982), and has been used extensively among Yoruba Nigerians where cut-off scores of ⩾11 had a *κ* agreement of 0.65 with psychiatrist diagnosed depression (Sokoya and Baiyewu, 2003).

### Main findings in relation to late-life MDD

#### High prevalence and incidence

Epidemiological studies of late-life depression in HICs (Thielke *et al*., 2010) as well as in LMICs (Guerra *et al*., 2016) report widely varying prevalence estimates. Community-based studies using standardised criteria such as the ICD 10 and DSM IV for clinically significant depression report an average prevalence of 3% (Goncalves-Pereira *et al*., 2019). Rates of late-life depressive episodes (Guerra *et al*., 2016) and MDD (Bromet *et al*., 2011) are observed to be higher in LMICs than in HICs, and in nearly all cases, the prevalence of clinically significant late-life depression is higher for women than for men and in rural locations than urban centres (Bromet *et al*., 2011; Guerra *et al*., 2016). By global standards, the ISA recorded high prevalence and incidence of late-life MDD. Lifetime and 12-month prevalence estimates were 26.2% [95% confidence interval (CI) 24.3–28.2] and 7⋅1% (95% CI 5.9–8.3), respectively (Gureje *et al*., 2007). Mean age at onset of MDD was 51 years for both men and women. The mean number of lifetime episodes was approximately two. When assessed in three follow-up waves over a period of 5 years (Ojagbemi *et al*., 2018*b*), the incidence rate of MDD of 120.9 per 1000 person years (95% CI 110.4–132.5) was found among 1394 persons who were free of lifetime MDD and dementia at baseline. Incidence rates were 94.7 (95% CI 82.5–108.7) and 153.8 (95% CI 136.3–173.6) per 1000 person years in men and women, respectively (Ojagbemi *et al*., 2018*b*).

#### Social nature of risk factors

Late-life MDD is consistently associated with economic, health and lifestyle factors in HICs (Goncalves-Pereira *et al*., 2019). These factors have also been significantly linked with depression in older adults living in LMICs (Lotfaliany *et al*., 2019). The risk factors for MDD in the ISA were mainly social in nature. For example, prevalent MDD was associated with poor social engagement (Ojagbemi and Gureje, 2019*a*), loneliness (Ojagbemi and Gureje, 2019*a*) and increasing level of urbanisation in the place of residence (Gureje *et al*., 2007) with odds ratios (OR) of 3.1, 2.3 and 1.4, respectively. Overall, poor social network was a key modifiable risk factor for incident MDD (Gureje *et al*., 2011; Ojagbemi *et al*., 2018*b*). Specifically, living in a rural location and having no regular contact with family (both of which can be regarded as indicators of poor social network) at baseline were associated with subsequent onset of MDD (Gureje *et al*., 2011; Ojagbemi *et al*., 2018*b*). One plausible interpretation of this finding is that the migration of younger family members to areas with better economic opportunities may be reducing the social network of elderly persons living in affected communities. Those who are unable to maintain regular contacts with migrated family members may be especially at risk of MDD.

The well-known relationship between low economic position and late-life depression in HICs (Goncalves-Pereira *et al*., 2019) was not replicated in the ISA with the use of asset-based measurement of economic status, a commonly used indicator of wealth in low-income settings (Ferguson *et al*., 2003). Nevertheless, incident MDD was associated with a lifetime of unskilled occupation in men (Ojagbemi *et al*., 2018*b*). Perhaps, this finding suggests that composite indices of economic position such as lifetime occupational attainment, which embraces education, longer term income, social status and potential for asset acquisition over-time, may be more stable and sensitive to capture longer term economic effect of late-life depression in SSA and other LMIC settings. For example, in many SSA locations with large subsistent farming populations, less stable measures of economic position may exhibit seasonal fluctuations. The health advantages of longer term and relatively stable economic status may accumulate over the life-course (Mensah and Hobcraft, 2008) and lead to a significant protective effect against the onset of MDD in old age (Kim and Durden, 2007; Lahelma *et al*., 2019).

#### Outcomes of late-life MDD

Similar to reports from both HICs (Goncalves-Pereira *et al*., 2019) and LMICs (Guerra *et al*., 2016), ISA participants with MDD had impaired quality of life and functioning in home, work and social roles. These outcomes worsened with increasing MDD symptoms severity (Gureje *et al*., 2007). Also, in pair-wise comparisons with arthritis, spinal pain, systemic hypertension, asthma and diabetes mellitus, late-life MDD in the ISA was associated with greater disability burden than the listed health conditions (Gureje *et al*., 2008). Specifically, approximately 47.2% of ISA participants with late-life MDD were rated as having a severe disability (Gureje *et al*., 2008). Conversely, severe disability among those with systemic hypertension, chronic spinal pain and arthritis was 25, 24.2 and 20.6%, respectively (Gureje *et al*., 2008). Late-life MDD comorbidity with the listed conditions was also very high at 61.7% overall (Ojagbemi *et al*., 2017*a*), with angina (28.2%) and uncorrected vision impairment (24.7%) (Ojagbemi *et al*., 2017*a*) standing out as the most comorbid health conditions with late-life MDD.

Access to MDD treatment rates was low (Gureje *et al*., 2007), and only about 37% of ISA respondents with a lifetime history of MDD received any treatment (orthodox, traditional or religious). Low economic status and residence in a rural area were associated with lack of treatment. For those who received these treatments, there was a mean delay of 5 years from the onset of MDD to receipt of first treatment (Gureje *et al*., 2007).

Symptomatic recovery from late-life MDD was possible, but this was commonly associated with persisting and worsening course of functional disability (ADL). In mixed-effect linear regression models constructed across 5 years of observation and adjusted for the effect of age, gender and socioeconomic status, ADL worsened in both respondents who recovered symptomatically from MDD [coefficient (*β*) = 1.0, 95% CI 0.2–1.8] and those who failed to achieve symptoms recovery (*β* = 2.3, 95% CI 1.6–3.0) (Ojagbemi *et al*., 2018a).

## Implication, recommendations and future directions

The ISA findings in relation to late-life MDD have important implications especially in the context of on-going social transitions in Nigeria and SSA. For example, changes that lead to attenuation of traditional supportive networks may be stripping older people in the setting of the ISA of protective buffers against depression. Previously, traditional gender roles were fairly well defined in the Nigerian contexts. Men are known to work outside the home and women to take up the role of ‘home making’ and provide emotional support to vulnerable family members (Adeleye *et al*., 2011). Changes that encourage women, who are the most common caregivers, to find jobs outside the home have the unintended consequence of exposing older persons in these settings to social isolation and its effects (especially depression) (Ojagbemi and Gureje, 2019*a*; Ojagbemi and Gureje, 2019*b*).

Results suggesting high prevalence and incidence, as well as poor access to late-life depression treatments in Nigeria, a situation that is likely to be applicable to other countries in SSA, highlight the public health challenge posed by MDD in the sub-region. Health systems in SSA are highly focused on physical health conditions, especially conditions with acute presentations, and less likely attuned to provide chronic care. In these circumstances, older people with mental health conditions are particularly disadvantaged in the competition for the scarce healthcare resources. Given the projected rapid increase in the population of older people in SSA, the common focus on acute physical health conditions, to the neglect of mental health, may become even more pronounced as greater demands are placed on the weak healthcare systems as a result of rapidly growing populations. To stem the neglect of older people's mental health in SSA, it is important for healthcare policies to specifically pay attention to the detection and treatment of depression and other mental health problems in late-life in primary health care settings.

The findings of the ISA suggesting that the disability associated with late-life depression persists despite symptomatic recovery also have implications for policy as well as for needed guidelines for the management of both depression and disability in older people. For example, future guidelines for the treatment of late-life MDD could consider a programme of rehabilitation for any resulting functional disability.

## Conclusion

The ISA provides ground-breaking information about late-life depression in SSA. Many of the findings are currently the basis for the design of clinical trials of tailored interventions for late-life MDD in SSA. The ISA opens up opportunities for further explorations of the data, leads for future research and avenues for collaborations that offer the prospect of advancing our knowledge of factors that shape the lived experience of depression in later life.
